# Mini-sternotomy for direct right atrial dialysis catheter placement in a child with central venous occlusion

**DOI:** 10.1007/s00467-025-07104-6

**Published:** 2025-12-09

**Authors:** Maria Nucera, Sibylle Tschumi, Matthias Siepe

**Affiliations:** 1 Department of Cardiac Surgery, Centre for Congenital Heart Disease, Inselspital, Bern University Hospital, University of Bern, 3010 Bern, Switzerland; 2 Department of Pediatrics, Pediatric Nephrology, Inselspital, Bern University Hospital, University of Bern, Bern, Switzerland

**Keywords:** Pediatric hemodialysis, Vascular access, Central venous occlusion, Right atrial catheter

## Abstract

We report the case of a pediatric patient with kidney failure and bilateral central venous occlusion, in whom conventional dialysis access was no longer feasible. Following multidisciplinary evaluation, a dialysis catheter was surgically inserted directly into the right atrium via a lower mini-sternotomy. A custom-made bovine pericardial conduit was used to facilitate extrathoracic tunnelling and protect the catheter. The procedure was technically successful, and the catheter remained functional postoperatively. This case highlights a viable salvage option for dialysis access in children with exhausted vascular access sites.

**Supplementary Information:**

The online version contains supplementary material available at 10.1007/s00467-025-07104-6.

## Background

Establishing and maintaining reliable vascular access remains a significant challenge in pediatric patients undergoing long-term hemodialysis. Repeated central venous catheter placements frequently lead to progressive thrombosis and eventual occlusion of major central veins, thereby limiting conventional access sites.

We report a rare case of direct right atrial catheter placement via lower mini-sternotomy in a child with complete occlusion of both the superior and inferior vena cava.

## Case presentation

The patient was a 5-year-old boy (dry weight 13.1 kg (*P* < 3, −2.79 SDS), height 96 cm (*P* < 3, −3.29 SDS)) with kidney failure since birth on chronic hemodialysis. The child had previously undergone a kidney transplantation, with a prompt graft failure due to arterial vascular occlusion and consecutive bleeding. Following graft loss, the patient returned to hemodialysis therapy, since peritoneal dialysis was not successful anymore after multiple abdominal interventions. Multiple central venous catheter placements over time had led to complete occlusion of both the superior and inferior vena cava. An endovascular attempt to recanalize the occluded vessels was unsuccessful. With no remaining conventional central venous access options and retransplantation not feasible due to persistent preformed antibodies despite desensitization attempts including immunoadsorption, rituximab, and IVIg, as well as peritoneal dialysis being unsuitable after multiple abdominal surgeries, a multidisciplinary team decided to proceed with surgical placement of a dialysis catheter directly into the right atrium.

### Surgical procedure

**Fig. 1:**
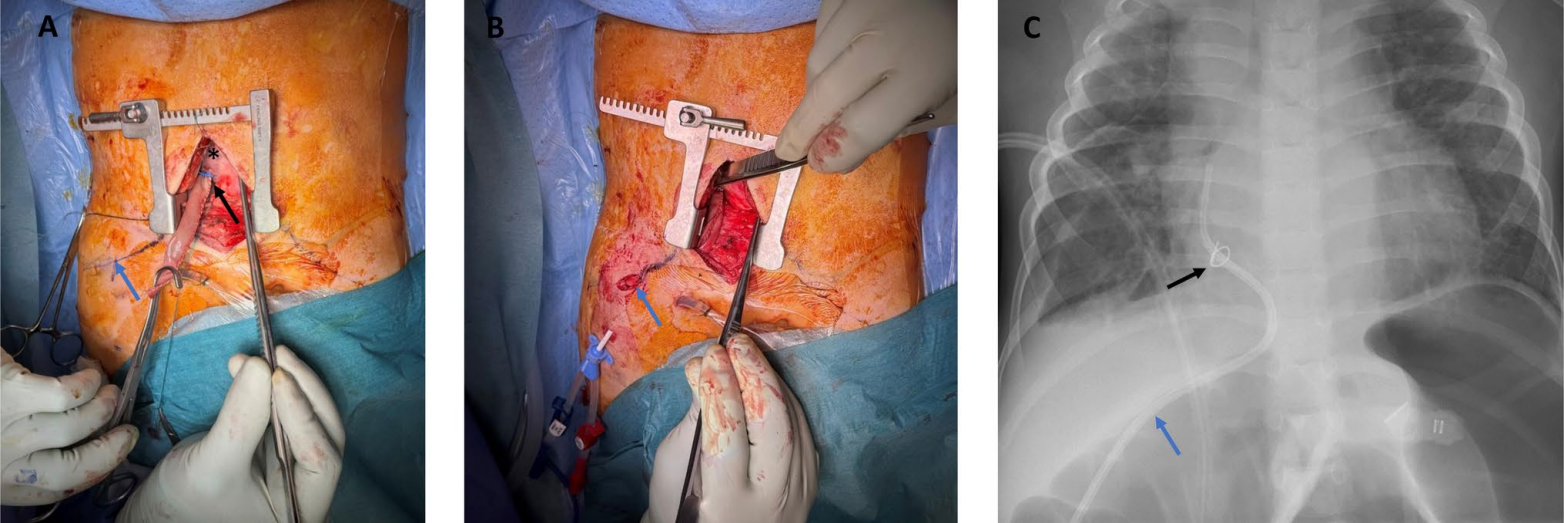
**A**, **B** Intraoperative image showing the pericardial tube with the radiopaque marker (black arrow), the right atrium (*), and the access sites: the lower mini-sternotomy and the subcostal incision (blue arrow). **C** Chest X-ray image showing the dialysis catheter with the radiopaque marker (black arrow)

A lower mini-sternotomy was carried out. After opening the pericardium, the right atrium was exposed. A tangential clamp was applied, and a longitudinal incision of approximately 1 cm was made. An 8 × 3 cm bovine pericardial patch was fashioned into a tube and sutured to the atrial incision, creating a direct conduit. A radiopaque marker was attached at the atrial entry site for future imaging reference (Fig. 1A).

The pericardial tube was then tunnelled subcostally through a second incision to the right side of the chest (Fig. 1B). A guidewire was introduced through the pericardial tube into the right atrium. Over the guidewire, an 8-Fr dual-lumen cuffed dialysis catheter (HemoCathDouble Lumen 8 F × 18 cm) was advanced. Under fluoroscopic guidance and contrast injection, the catheter tip was positioned so that the distal end was directed toward the superior vena cava, while the proximal portion resided approximately 1 cm within the right atrium. Initially, flow through the catheter was suboptimal, due to overly tight fixation. After adjusting, both lumens could be freely aspirated and flushed. The sternum and the incisions were closed and the catheter was securely fixed to the skin (Fig. 1C, Supplemental Video).

### Postoperative phase and follow-up

The postoperative course was initially uneventful, and the catheter remained fully functional with good flow (mean blood flow rate of approximately 95 ml/min = 7.2 ml/kg/min resp. 161 ml/m^2^/min).

However, 2 months later, during a routine dressing change, the catheter was accidentally transected.

The patient was taken back to the operating room. Through the original subcostal incision, the pericardial tube was re-identified and carefully dissected free. Using the Seldinger technique, a guidewire was first introduced through the lumen of the existing catheter prior to its removal. Subsequently, a new dialysis catheter was advanced and positioned successfully under echocardiographic guidance. Intraoperative testing confirmed proper catheter function, and the postoperative course remained stable thereafter. For another 6 months after the revision, the catheter is fully functional.

## Discussion

Central venous occlusion represents a significant and increasingly common challenge in pediatric patients undergoing long-term dialysis, particularly when repeated catheter placements have rendered conventional access sites unusable. In such complex clinical scenarios, alternative techniques must be considered to maintain life-sustaining dialysis therapy.

Preventive strategies are crucial to avoid exhaustion of vascular access. As recommended by Preka et al., early planning of vascular access, strict preservation of central veins, and a “catheter-last” approach — favoring arteriovenous fistulas over central venous catheters whenever possible — are key measures [1]. These strategies are particularly important in children with long expected dialysis duration and limited transplant options and are supported by both the European Society for Paediatric Nephrology Dialysis Working Group and KDOQI guidelines [2, 3].

When conventional access is exhausted, several alternative vascular access strategies have been described. Inston et al. provided a structured overview of options for “end-stage vascular access,” including translumbar or transhepatic central venous catheters, arterio-arterial grafts, and right atrial grafts [4]. Each approach carries specific risks and should be reserved for cases in which conventional options are no longer viable.

Direct right atrial catheterization, though rarely performed in children, emerges as a potential salvage option when all standard vascular access routes have been exhausted. While this approach is invasive and technically demanding, it offers a durable solution in select cases. In the present case, the use of a bovine pericardial tube as a conduit provided dual benefits: it served as a protective sheath and facilitated extrathoracic tunnelling of the catheter, thereby potentially reducing the risk of infection.

The existing literature on this technique remains limited, consisting mainly of isolated case reports in both pediatric and adult populations. Detering et al. reported the successful placement of a Hickman catheter via thoracotomy into the right atrium of an 11-year-old girl, which functioned effectively for parenteral nutrition [5]. Similarly, Negoi et al. described a case in which a tunnelled right atrial catheter remained functional for 14 months in an adult dialysis patient with no remaining vascular access, without any reported complications [6]. In a more recent review including 51 tunneled right atrial catheters in adult patients, Philipponnet et al. found acceptable patency rates and manageable complication profiles, supporting the feasibility of this technique in select cases [7].

These reports, along with the current case, underscore the feasibility of right atrial catheterization in highly selected patients. However, both the European Society for Paediatric Nephrology Dialysis Working Group and the National Kidney Foundation Kidney Disease Outcomes Quality Initiative emphasize that such procedures should be strictly reserved for patients with no other viable access options and should be carried out by experienced multidisciplinary teams [2, 3].

Limitations of this report include the short follow-up period, as the patient underwent surgery in March 2025. Longer observation will be necessary to assess long-term patency and potential complications.

In conclusion, while direct right atrial access should not be considered routine, it may provide a crucial lifeline in otherwise untenable situations.

## Conclusion

To our knowledge, this is the first case described using direct right atrial catheter placement using a bovine pericardial conduit via mini-sternotomy in a child. This approach may be considered a salvage option in pediatric dialysis patients with bilateral central venous occlusion.

## Summary

### What is new?

- Novel salvage access: direct right atrial catheter placement for dialysis in a pediatric patient with bilateral central venous occlusion.

## Supplementary Information

Below is the link to the electronic supplementary material.

**Supplemental video:** Presentation of the surgical procedure (MP4 69.5 MB).

## Data Availability

All relevant data are within the manuscript. Derived data supporting the findings of this case report are available from the corresponding author on request.
